# Role of Hydration and Amino Acid Interactions on the Ion Permeation Mechanism in the hNa_*V*_1.5 Channel

**DOI:** 10.1021/acs.biochem.4c00664

**Published:** 2024-12-17

**Authors:** Nuria Anguita-Ortiz, Juan J. Nogueira

**Affiliations:** † Department of Chemistry, Universidad Autónoma de Madrid, Calle Francisco Tomás y Valiente, 7, 28049 Madrid, Spain; ‡ IADCHEM, Institute for Advanced Research in Chemistry, Universidad Autónoma de Madrid, Calle Francisco Tomás y Valiente, 7, 28049 Madrid, Spain

## Abstract

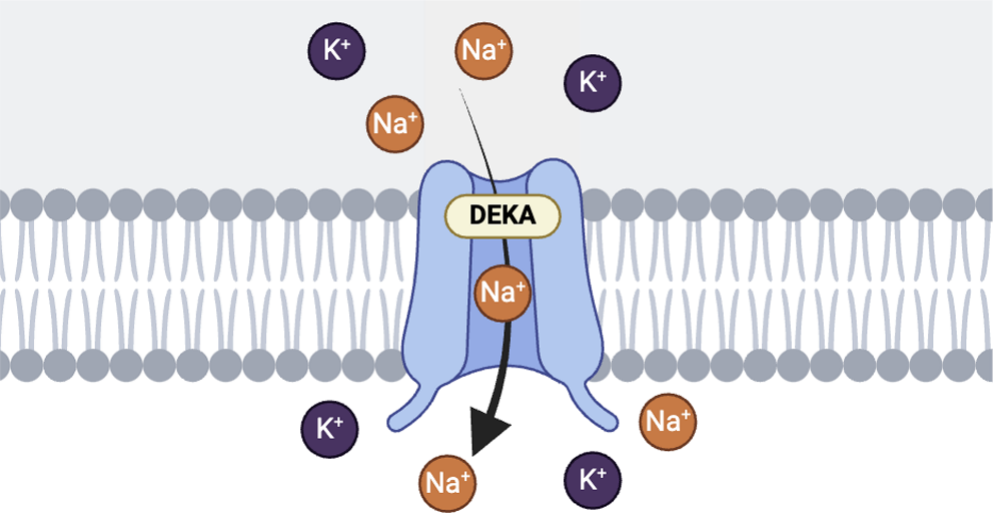

This study explores the ion selectivity and conduction mechanisms of the hNa_*V*_1.5 sodium channel using classical molecular dynamics simulations under an externally applied electric field. Our findings reveal distinct conduction mechanisms for Na^+^ and K^+^, primarily driven by differences in their hydration states when they diffuse close to the channel’s selective filter (DEKA) and extracellular ring (EEDD). The Na^+^ ions undergo partial dehydration in the EEDD region, followed by a rehydration step in the DEKA ring, resulting in longer retention times and a deeper free energy minimum compared to K^+^. Conversely, the K^+^ ions exhibit a continuous dehydration process, facilitating a smoother passage through these key regions. These results indicate that ion selectivity and conductance are primarily governed by solvation dynamics, which, in turn, depend on the interactions with key charged residues within the channel. Additionally, we show that the delicate energetic balance between the interactions of the ions with the protein residues and with their solvation shells during the dehydration and rehydration processes is not properly captured by the force field. As a consequence, the selectivity of the channel is not well described, indicating that more accurate computational models must be applied to simulate ion conduction through eukaryotic Na_*V*_ channels.

## Introduction

The physiological significance of voltage-gated sodium (Na_*V*_) channels is highlighted by their central role in regulating membrane excitability.^1−3^ The malfunction of this process is associated with a wide range of neurological, cardiovascular, muscular, and psychiatric disorders, such as epilepsy, arrhythmias, muscle paralysis, pain syndromes, and autism spectrum disorders.^4−6^ The precise response of Na_*V*_ channels to subtle alterations in membrane voltage, activating or deactivating within milliseconds, is crucial for essential functions such as synaptic transmission in neurons and rapid muscle fiber contractions. Consequently, they play a pivotal role not only in propagating electrical signals across nerve pathways but also in muscle activation, underscoring their significance in neurology and cardiology. Moreover, Na_*V*_ channels are targets for various natural toxins and medical treatments, such as blockers, which are widely used to treat cardiac arrhythmias and prevent epileptic seizures by modulating abnormal electrical activity.^7,8^ These channels are situated within the plasma membranes of excitable cells, such as neurons and skeletal and cardiac muscle cells, orchestrating the swift transit of sodium ions (Na^+^) across the membrane. They facilitate Na^+^ influx from the extracellular region—where the concentration is around 150 mM—to the cytoplasm, where levels are markedly lower, approximately 10 mM. This flux results in a net positive shift inside the cell, diminishing the membrane potential and initiating the depolarization process, which is critical for the rising phase of action potentials.^1,9,10^

To gain a comprehensive understanding of the conduction mechanism and the functional and structural diversity of sodium channels, it is essential to consider both the prokaryotic and eukaryotic versions. Prokaryotic Na_*V*_ channels are simpler, composed of four identical subunits, each composing of six transmembrane helices that together create a central pore and a voltage-sensing apparatus. This tetrameric structure, exemplified by channels such as NaChBac,^11^ is less adaptable than eukaryotic channels due to its single-domain composition, which limits flexibility in regulatory mechanisms and response to environmental changes. They feature a simple selectivity filter made up of four glutamate residues (EEEE).^7,12−15^

Molecular dynamics simulations have elucidated the ion conduction mechanisms of prokaryotic channels.^13,16,17^ Such simulations have been pivotal in studying ionic permeation and selectivity, providing a comprehensive visualization of the ion translocation process that aligns well with electrophysiological observations. For instance, studies focusing on the Na_*V*_Ms channel structure, which features an open cytoplasmic gate, have demonstrated continuous ion translocation across the membrane and have accurately predicted channel conductance.^12^

Eukaryotic channels, on the other hand, are formed from a single polypeptide chain that folds into four homologous domains, each containing six transmembrane segments.^18,19^ This allows for more sophisticated modulation and regulation, facilitated by multiple binding sites for cofactors and metabolic modulators, and by their larger size and structural complexity.^11^ In humans, nine distinct subtypes of voltage-gated sodium (Na_*V*_) channels are identified, ranging from Na_*V*_1.1 to Na_*V*_1.9.^5,19^ These channels are strategically located in various tissues, including the central and peripheral nervous systems, and in skeletal and cardiac muscles, with the hNa_*V*_1.5 subtype, studied in this work, being particularly significant in the cardiac function.^20^ Each subtype exhibits unique ion permeation characteristics and is regulated not only by its placement within the membrane but also by its interaction with auxiliary β subunits.

**Figure 1:**
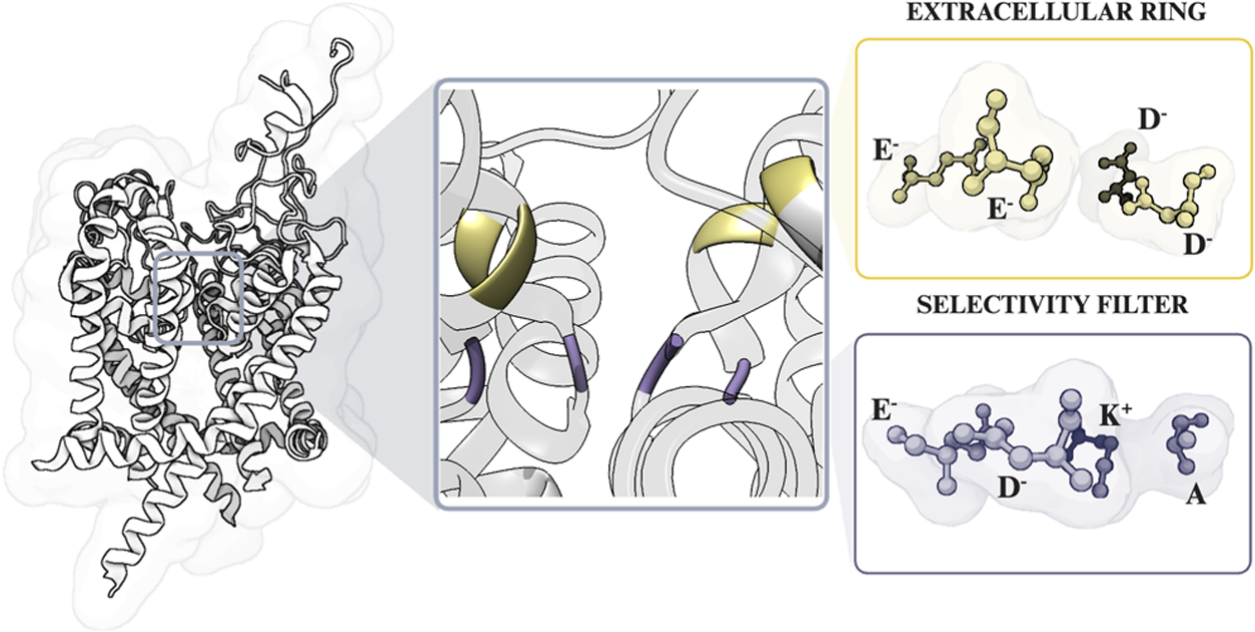
Representation of the pore structure of the voltage-gated sodium channel Na_*V*_1.5. SF is colored purple, indicating the amino acids of the DEKA motif, and the extracellular ring composed of EEDD residues is displayed in yellow.

Eukaryotic Na_*V*_ channels are distinguished by their uniquely asymmetric selectivity filter (SF), organized around four amino acids (DEKA) at the narrowest part of the channel, known as the constriction site, as shown in Figure 1. Composed of aspartate (D), glutamate (E), lysine (K), and alanine (A), this selective filter (DEKA) is strategically positioned to confer selectivity for Na^+^ ions over K^+^ or other ions, as observed experimentally.^21^ The lysine residue, located in domain III, is believed to be the critical amino acid responsible for the channel’s selectivity. This arrangement ensures not only high specificity but also efficiency in Na^+^ ion conduction, which is essential for the rapid propagation of action potentials.^22^ Additionally, these channels feature an extracellular ring of glutamate and aspartate residues (EEDD) at the channel’s entrance, as shown in Figure 1, enhancing ion attraction and further facilitating efficient electrical signal transmission.^7^ The functional cycles of these channels navigate through resting, active, and inactivated states, governed by the conformational dynamics of the voltage-sensing domains responding to shifts in the membrane potential. The rapid inactivation mechanism, prompted by a hydrophobic motif within the linker between the third and fourth domains, is essential for the successive generation of action potentials.

Given the abundance of data on prokaryotic channels and the relative lack of detailed analyses on their eukaryotic counterparts, this study is aimed at unraveling the ion conduction mechanism of the hNa_*V*_1.5 channel, whose operational dynamics are not fully understood. In this work, the conduction mechanism of hNa_*V*_1.5 is investigated by means of conventional and electric-field-biased classical molecular dynamics simulations. The key factors involved in ion conduction events, namely ion dehydration and interactions between ions and the SF, are analyzed in detail. Moreover, the performance of the force field to describe ion selectivity is discussed.

## Materials and Method

### System Setup

**Figure 2:**
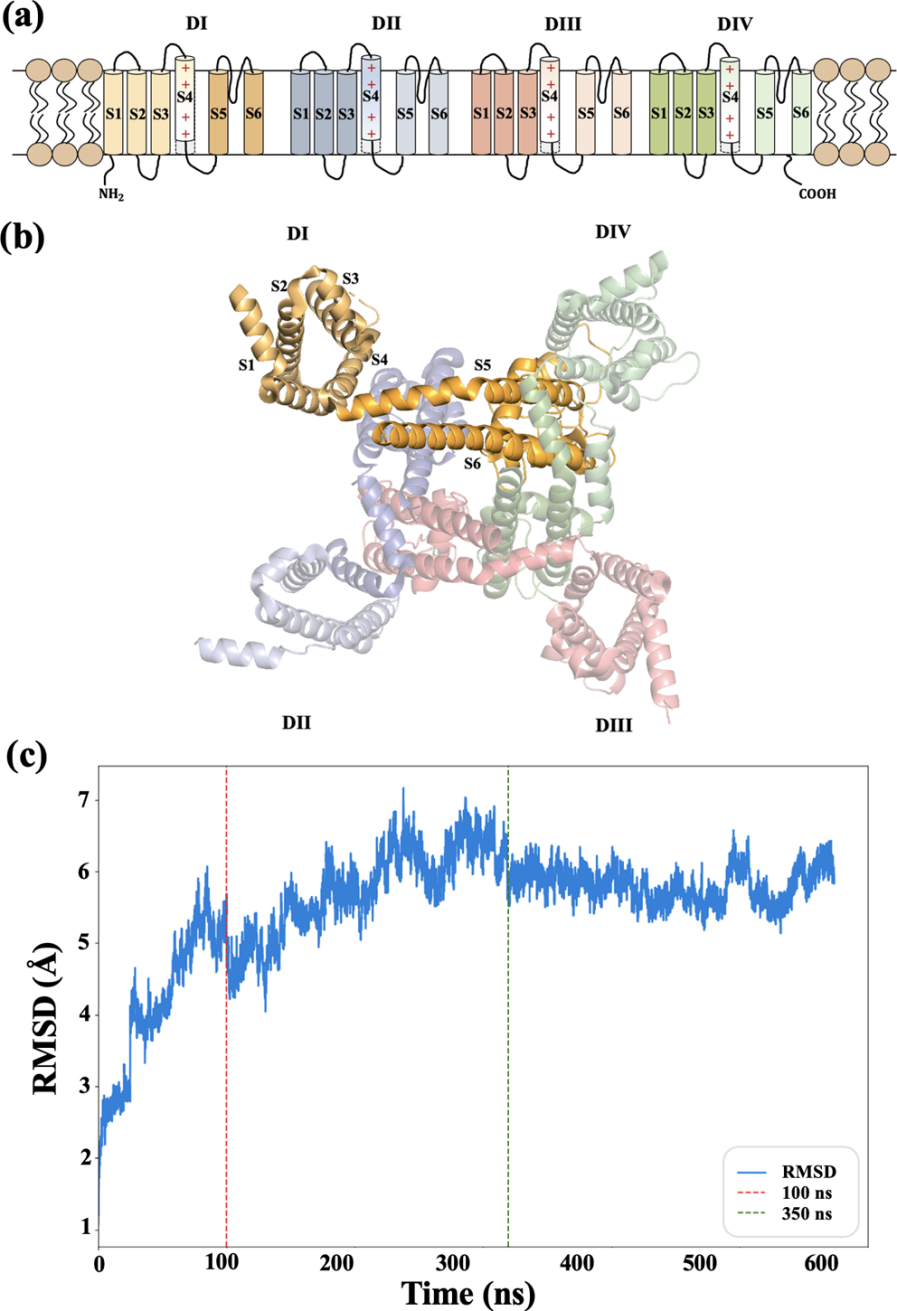
(a) Schematic diagram of the Na_*V*_1.5 channel displaying the four domains (DI-DIV) and their transmembrane segments (S1–S6). The positively charged segments (S4) are marked with “+”, indicating their role in VSD. (b) Bottom-view three-dimensional representation of the Na_V_ channel, where each domain is colored differently (DI in yellow, DII in pink, DIII in green, and DIV in light blue) to highlight the spatial organization and orientation of the S1–S6 segments within the membrane structure. (c) Root mean squared displacement (RMSD) of the nonhydrogen atoms of the protein along the simulation. The dashed vertical line represents the 100 ns simulation without the application of an electronic field. The dashed green line is placed after 250 ns of biased simulation, from which convergence has been achieved.

Protein coordinates were obtained from the Protein Data Bank under the entry 7FBS,^20^ resolved by electron microscopy at a 3.40 Å resolution without lipid components. Initially crystallized in its open conformation, the structure underwent a preliminary refinement using PyMol^23^ to excise all water molecules and extraneous ligands. Additionally, the transmembrane segments S1–S4, corresponding to the voltage-sensing domains (VSD) across DI, DII, DIII, and DIV, were removed, retaining only the S5 and S6 segments that constitute the channel’s pore (Figure 2a,2b). This modification was necessary due to the large size of the system, which would increase considerably the computational time. In this way, we focused on the channel pore and its SF, which are essential for the study of ion conduction mechanisms.

The system was described by the CHARMM36m force field,^24^ which was chosen for its precise modeling of interactions between proteins and lipids in biomimetic environments. The CHARMM-GUI web-based graphical user interface^25^ assisted in aligning the protein along the *Z*-axis with the aid of the orientations of proteins in membranes (OPM) database,^26^ subsequently determining the dimensions of the simulation box. For titratable residues, we assigned the protonation states using the standard conditions provided by CHARMM-GUI, which assume physiological pH. The protocol applied by CHARMM-GUI,^27^ using the Bilayer Builder Input Generator, initiates by selecting a rectangular box, setting the *Z*-axis length according to a predefined water layer thickness of 22.5 Å, necessary for solvating the system. The *X* and *Y* dimensions were determined based on the lipid component ratios, resulting in surface areas of 7581.3 Å^2^ (upper leaflet) and 7991.1 Å^2^ (lower leaflet), respectively. The protein was embedded in a pre-equilibrated palmitoyloleoylphosphatidylcholine (POPC) lipid bilayer, commonly found in biological membranes, chosen for both the upper and lower leaflets, which facilitated an initial estimate of the *X* and *Y* dimensions. The initial system dimensions were 100.094422 Å × 100.094422 Å × 121.693318 Å. Moreover, the electrolytes NaCl and KCl, described by the Joung parameters,^28^ were integrated at a concentration of 150 mM within TIP3P water^29^ to replicate physiological conditions. In the initial system setup, sodium (Na^+^) and potassium (K^+^) ions were incorporated simultaneously within the same simulation box, creating a mixed system from the beginning. Consequently, the molecular dynamics simulation was performed under conditions where both types of ions coexist, providing a comparative assessment of their behaviors within the same structural framework. This approach was chosen to evaluate the differences in the permeability and behavior of the ions under identical initial conditions. The Na^+^ and K^+^ ions were initially positioned randomly within the solvent region to approximate physiological concentrations. Upon completion of the system setup, inputs for NAMD^30^ were generated, readying the simulation for execution. This streamlined setup, utilizing a single lipid type, allows for an exploration of the fundamental mechanisms of ion transport and channel behavior without the complexity required for a fully detailed and more realistic lipid environment. Such an approach has proven successful in analogous studies of prokaryotic voltage-activated sodium and potassium channels.

### Classical Molecular Dynamics

The systems were initially minimized by the conjugate gradient and line search methods, extending over 10000 steps to ensure a robust and stable starting configuration. Then, the system underwent a thermal equilibration and was segmented into three distinct stages within the NVT ensemble. Each stage utilized a time step of 1 fs, with the system’s temperature progressively increasing from 0 to 100 K in the first phase, from 100 to 200 K in the second, and culminating at 303.15 K in the final phase. The entire heating protocol spanned 0.75 ns, with each phase allocated 0.25 ns. During the initial phase of equilibration, constraints were applied to the α carbons of the protein, with a force constant initially set at 10.0 kcal/(molA^2^) in the first heating step, which was reduced to 5 kcal/(molA^2^) and 2.5 kcal/(molA^2^), in the second and third steps, respectively. Temperature regulation was controlled using the Langevin thermostat,^31^ with a collision frequency of 1.0 ps^–1^.

After the sample was heated, an equilibration simulation was evolved in the NPT ensemble, allowing changes in the volume of the simulation box to achieve an optimal system density. The constraints were gradually relaxed from 1 to 0.5 kcal/(molA^2^) and finally to 0.1 kcal/(molA^2^), before being completely removed for the production phase. Throughout these stages, a time step of 2 fs was maintained, resulting in an equilibration period of 1.5 ns. Pressure control was handled by the Nose-Hoover Langevin piston,^32^ setting a target pressure of 1.01325 bar equivalent to one atm, with a piston oscillation period of 50 fs and a decay time of 25 fs. During the entire protocol, the electrostatic interactions were computed by the Particle-Mesh Ewald (PME) method,^33^ utilizing a grid spacing of 1.0 Å, complemented by a 12 Å cutoff and a 10 Å switching distance for nonbonded interactions, ensuring both computational efficiency and accuracy. Bonds involving hydrogen atoms were effectively restrained using the SHAKE algorithm.^34^ The production phase began with an initial 100 ns simulation in the NPT ensemble without an external electric field designed to equilibrate the system under physiological conditions. Following this, an additional 500 ns simulation was performed, this time applying an external electric field of 1.0 V along the *Z*-axis to enhance the ion mobility and increase the occurrence of conduction events. This electric field is larger than the one occurring in the cell membrane, usually around 70–90 mV, but it is necessary to accelerate the ion mobility through the channel pore, enriching the data set on ion permeation events and thereby facilitating a thorough and consistent analysis. Potential-biased molecular dynamics simulations have been successfully applied in the past to investigate the ion conduction of bacterial channels.^7,13,16,17^ Additionally, fixed constraints were applied to α-Carbon atoms of residues ALA414, LEU938, ILE1472, and ILE1770 in the channel gate throughout the entire simulation to ensure structural stability in the absence of the voltage-sensing domains. This approach was intended to preserve the channel in an open conformation, minimizing conformational changes that could potentially affect the ion conduction and selectivity. While the removal of the voltage-sensing domains could introduce artifacts, prior studies indicate that such modifications generally do not significantly affect the pore’s functional properties regarding ion permeation and selectivity.^7,13,16^

## Results

The analysis has been performed using in-house developed Python scripts for the converged 250 ns of the NPT production phase with an external electric field from a total of 500 ns that were simulated. These 250 ns were selected because they represent the portion of the simulation that is well converged, as shown in Figure 2c, where the root mean squared displacement of the nonhydrogen atoms of the protein has reached a constant value. The application of an external electric field was aimed at enhancing ionic mobility, thus enabling a more statistically meaningful analysis of ion transport. The current analysis was conducted and segmented into two sections. The first section computes the number of conduction events for the sodium and potassium ions through the channel and the free energy profiles. This is followed by an analysis of the dehydration of the ions and the interaction with the residues of the selective filter (DEKA) and the extracellular ring (EEDD) immediately above the DEKA that coordinates the ions along the transport pathway. The general goal is to unveil the role of water and protein residues during ion conduction events and assess the performance of the force field in describing ion conduction.

### Ion Flux and Free Energy Profile

**Figure 3:**
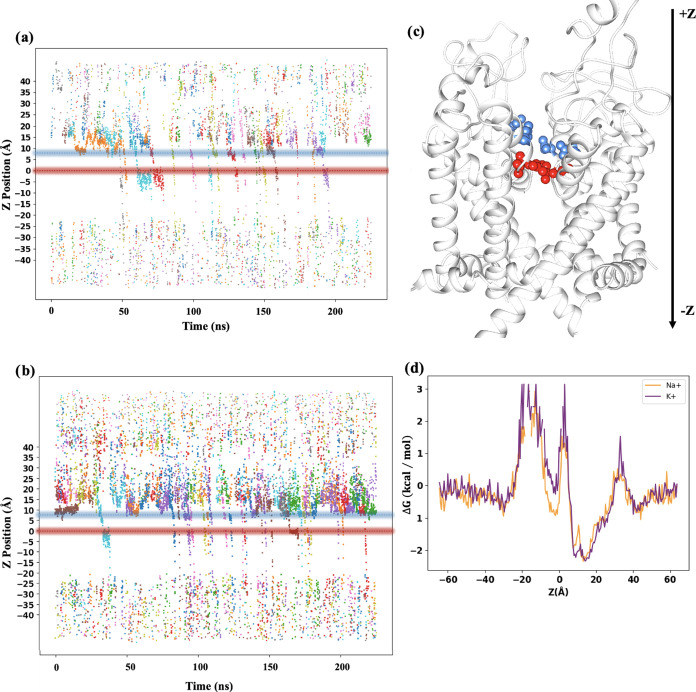
Ion permeation profiles for (a) Na^+^ and (b) K^**+**^ along the *Z*-axis in Å over time in nanoseconds (ns). (c) Representation of the EEDD residues (blue spheres) and DEKA residues (red spheres) in the protein. (d) Relative binding free energies (△*G*(*z*)) for Na^+^ and K^+^ along the permeation pathway.

Ionic translocation events through a Na_*V*_ channel are expected to be more abundant for Na^+^ than for K^+^ ions. Nevertheless, this expected trend is not reflected in our simulations. The number of conduction events for Na^+^ (Figure 3a) is lower compared to K^+^ (Figure 3b). Specifically, over the last 250 ns of simulation, 26 conduction events were observed for K^+^, whereas only 17 events were recorded for Na^+^, which are represented in the figure by colored dots migrating from the extracellular region (*Z* = 20 Å) to the intracellular region (*Z* = −20 Å). Therefore, the first relevant conclusion is that the employed force field does not provide quantitative accuracy since it is unable to describe ionic selectivity. However, our simulations properly highlight the importance of the selectivity filter (DEKA, *Z* = 0–5 Å) represented by red spheres in Figure 3b and of the extracellular ring (EEDD, *Z* = 5–7 Å) represented by blue spheres, as discussed in previous investigations.^7,16^ Therefore, although the simulations presented here may lack precise quantitative predictability, they nonetheless provide valuable qualitative insights into the mechanisms underlying ionic permeation through the channel.

The analysis displayed in Figure 3a,b shows that the ions have been trapped in the DEKA and EEDD rings for some time. In particular, it was found that the average retention times for Na^+^ and K^+^ ions in the EEDD region were 1.78 and 1.04 ns, respectively. In the case of the DEKA ring, the retention time difference between both ions is larger: 1.46 ns for Na^+^ and 0.68 ns for K^+^. It is important to highlight that the individual values of these times are not meaningful because of the unrealistically large potential we are applying to enhance the dynamics. However, the comparison between the Na^+^ and K^+^ retention times provides valuable information regarding the dynamics. The retention times correlate well with the relative free energy profile, *G*(*z*), displayed in Figure 3d, which is computed as
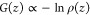
1The free energy profiles were obtained from the ion population density distribution, ρ(*z*), along the *Z*-axis, calculated in bins of 0.5 Å. Specifically, ρ(*z*) is obtained by counting the number of times the ions are located in a particular bin along the *Z*-axis through the simulation. These distributions were normalized and then converted into free energy profiles by the previous equation. The high electric field applied during the simulations might artificially influence the shape of the free energy profiles. However, the unbiasing of the simulations is not needed for comparison purposes of both profiles, which were generated under the same conditions. Overall, the free energy profiles for both ions exhibit similar characteristics. They present a minimum of −2 kcal/mol at around *Z* = 10 Å, which corresponds with the EEDD ring. Then, the ions must overcome an energy barrier of 5 kcal/mol to reach the DEKA ring at the selectivity filter (*Z* = 0 Å). Here is where the only noticeable difference between Na^+^ and K^+^ is found. The energy minimum is 1 kcal/mol deeper for Na^+^ than that for K^+^. This correlates well with the larger retention time for Na^+^ (1.46 ns) than for K^+^ (0.68 ns). The presence of this deeper minimum for Na^+^ could be a consequence of two factors: (i) more favorable intermolecular interactions with the residues of the channel or (ii) a slow hydration/dehydration process that retains the Na^+^ ion in the channel, lowering the free energy. In the following section, both factors are investigated. Coming back to the free energy profile, in the last conduction mechanism step, the ions must overcome a second energy barrier to transit from the DEKA ring to the pore of the channel.

### Ion/Protein Interactions and Dehydration

The deeper energy minimum found for Na^+^ than for K^+^ in Figure 3d can be a consequence of strong favorable interactions with the residues of the channel and, in particular, with the DEKA residues. In the following, we will analyze the interactions between the ions and the EEDD and DEKA rings in terms of coordination numbers. Thus, a larger coordination number of a particular amino acid indicates that this residue interacts more favorably with the ion, while a low coordination number probes the presence of repulsive interactions or the absence of interaction. Here, we define that an amino acid coordinates an ion if the ion is found within a specific coordination radius, measured from the oxygen atoms of the carboxyl groups in the residue. This coordination radius was determined iteratively by analyzing the average coordination number of the ions across increasing cutoff distances. The chosen coordination radius corresponds to the point where the coordination number becomes constant, indicating the optimal distance for capturing relevant ion-residue interactions.

**Figure 4:**
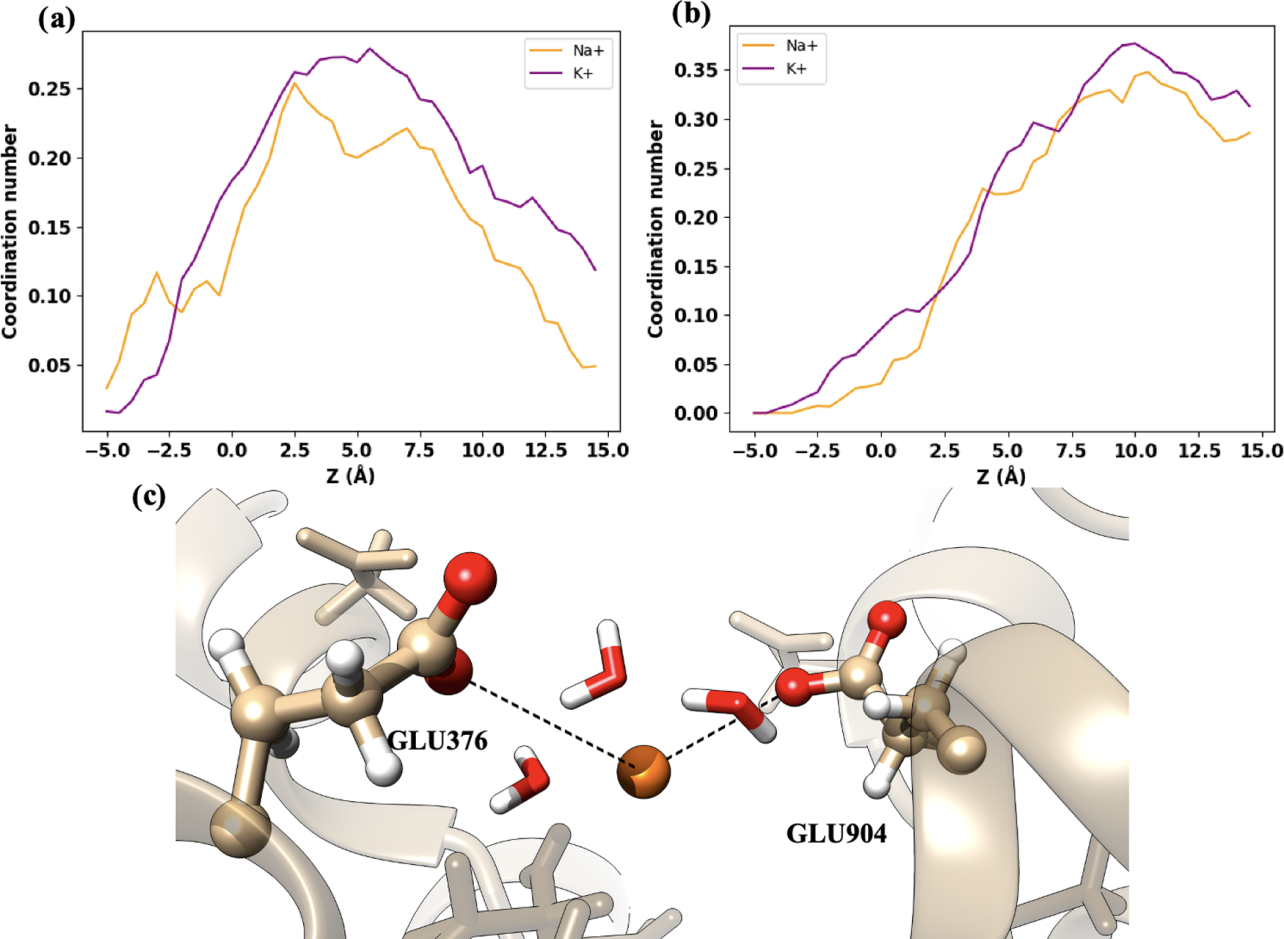
Ion coordination by GLU in the EEDD region. (a, b) Coordination numbers for Na^+^ (orange) and K^+^ (purple) with GLU376 and GLU904, respectively. (c) Representation of Na^+^ interacting with GLU376 and GLU904.

**Figure 5:**
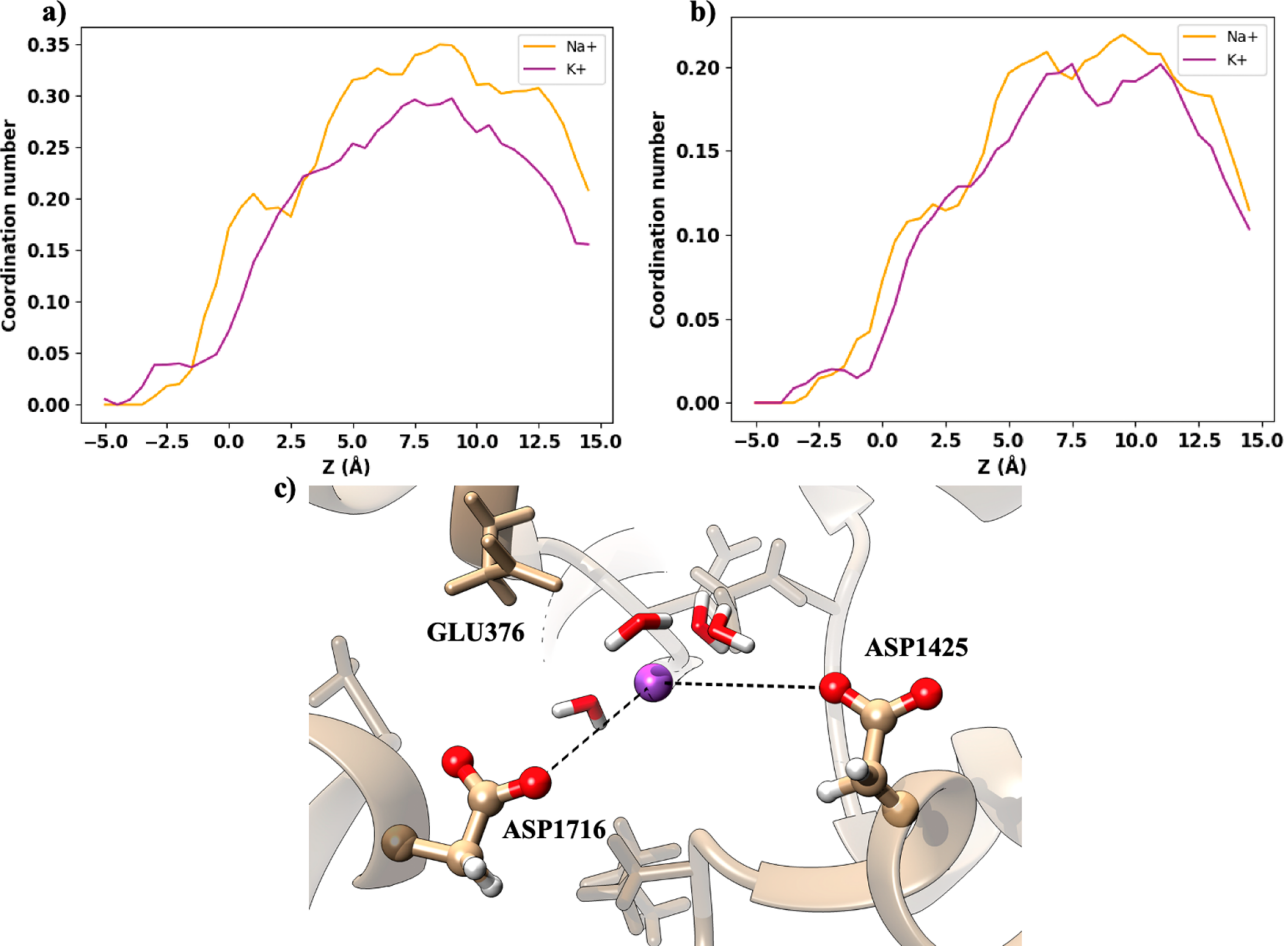
Ion coordination by ASP in the EEDD region. (a, b) Coordination numbers for Na^+^ (orange) and K^+^ (purple) with ASP1425 and ASP1716, respectively. (c) Representation of Na^+^ interacting with ASP1425 and ASP1716.

The EEDD ring consists of four negatively charged amino acid residues, creating a highly interactive environment for both the Na^+^ and K^+^ ions. Figure 4a,b shows that the coordination with the glutamates, GLU376 and GLU904, is similar for both ions. In the same way, the coordination numbers of the two aspartates, ASP1716 and ASP1425, are also virtually the same for Na^+^ and K^+^, as can be seen in Figure 5a,b. This agrees well with the similarity of both free energy profiles in this region of the channel, where both ions have stronger interactions with the negatively charged EEDD ring. Representative snapshots of Na^+^ and K^+^ interacting with the EEDD residues are displayed in Figures 4c and 5c, respectively. It can be seen that the ion solvation shell at the EEDD has been partially broken for both ions, as will be quantified later, to maximize the attractive interactions with the negatively charged amino acids.

**Figure 6:**
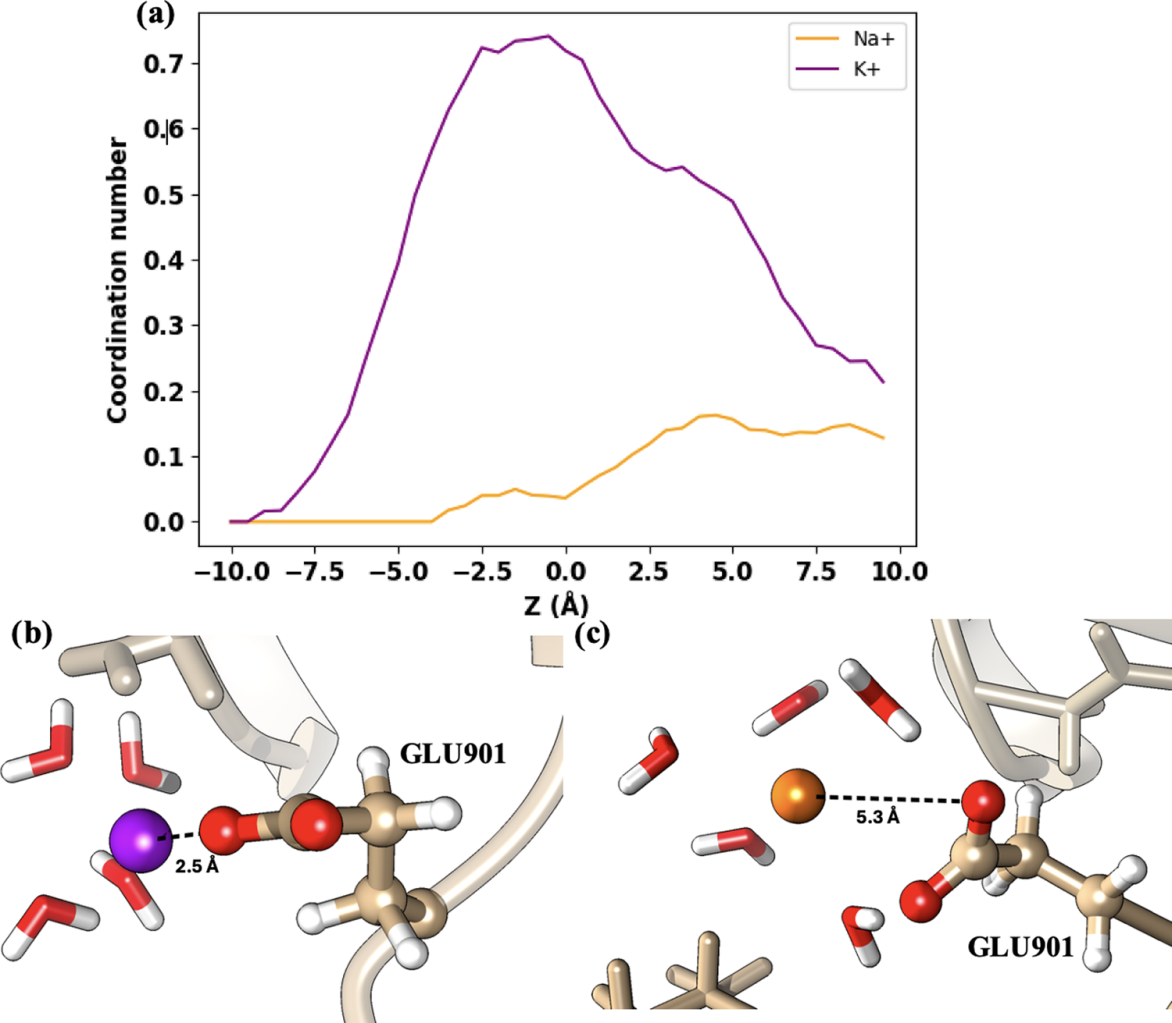
Ion coordination by GLU901 in the DEKA region. (a) Coordination numbers for Na^+^ (orange) and K^+^ (purple) along the *Z*-axis in the DEKA region. (b) Visualization of a potassium ion positioned closely (2.5 Å) to GLU901. (c) Sodium ion shown at a greater distance (5.3 Å) from GLU901.

**Figure 7:**
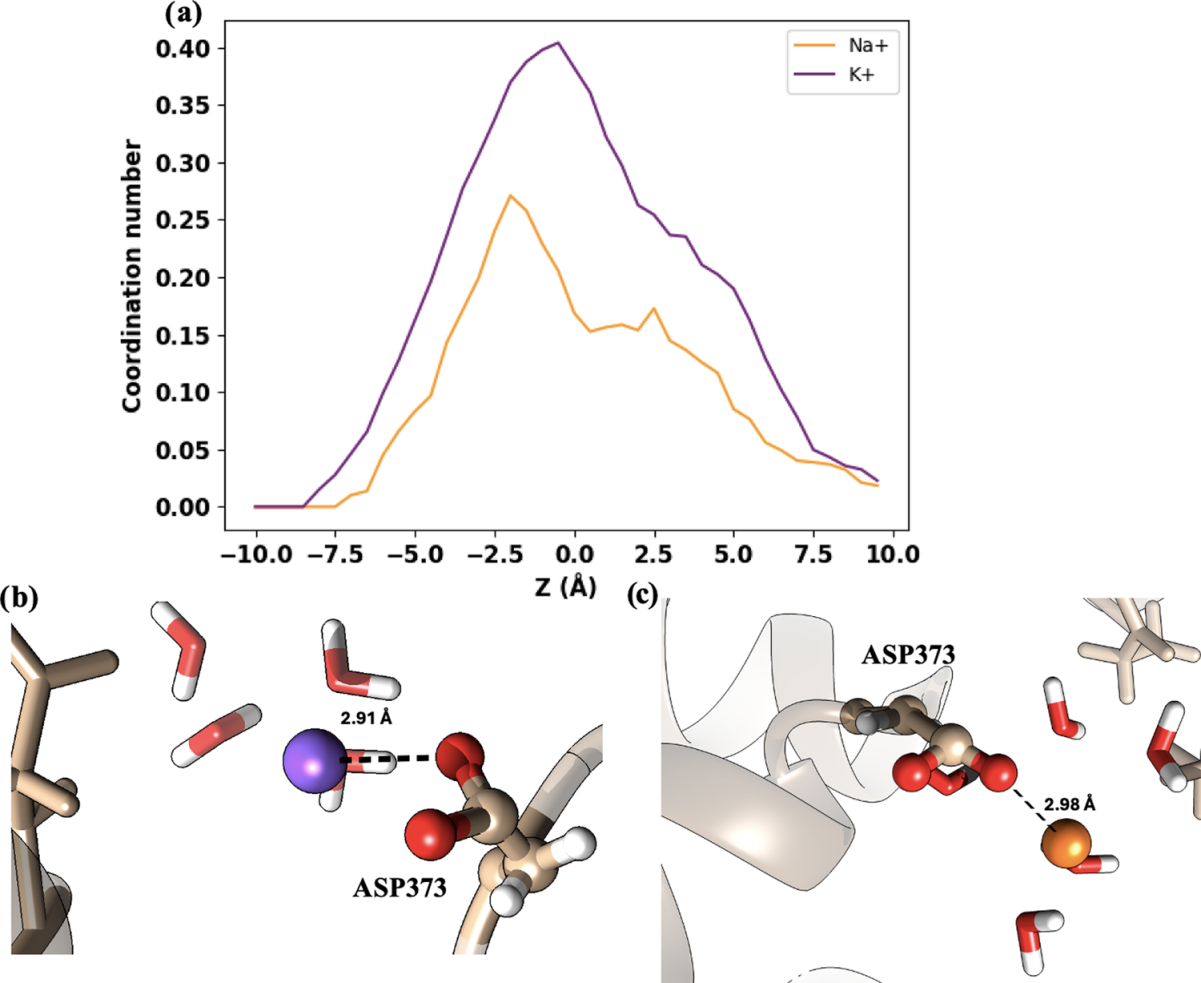
Ion coordination by ASP373 in the DEKA region. (a) Coordination numbers for Na^+^ (orange) and K^+^ (purple) along the *Z*-axis in the DEKA region. (b) Visualization of a potassium ion positioned closely (2.91 Å) to ASP373. (c) Sodium ion is shown at a greater distance (2.98 Å) from ASP373.

The DEKA motif at the SF has two negative (glutamate and aspartate) and one positive (lysine) amino acid, resulting in a net charge of −1, much smaller than the −4 charge of the EEDD ring. Therefore, the interactions with the ions might be less attractive, or even repulsive, at DEKA. This is indeed the case for the Na^+^ ion. Figures 6a and 7a show that the maximum coordination numbers of Na^+^ are 0.1 and 0.25 when the ion interacts with GLU901 and ASP373, respectively. However, the situation is different for the K^+^ ion, which has larger coordination numbers with both residues. This can also be seen in the representative snapshots displayed in Figures 6b,c and 7b,c, where the separation between Na^+^ and the amino acids is larger than that between K^+^ and the amino acids. Therefore, the coordination scenario suggests that the interactions with the DEKA motif are more favorable for K^+^ than for Na^+^. This is in apparent contradiction with the free energy profile shown in Figure 3d, where a deeper minimum and a larger retention time at the DEKA region is observed for Na^+^. Therefore, there must be another factor that explains the lower free energy for Na^+^ at the DEKA. This factor is found when the water solvation sphere of the ions is analyzed along the conduction pathway.

**Figure 8:**
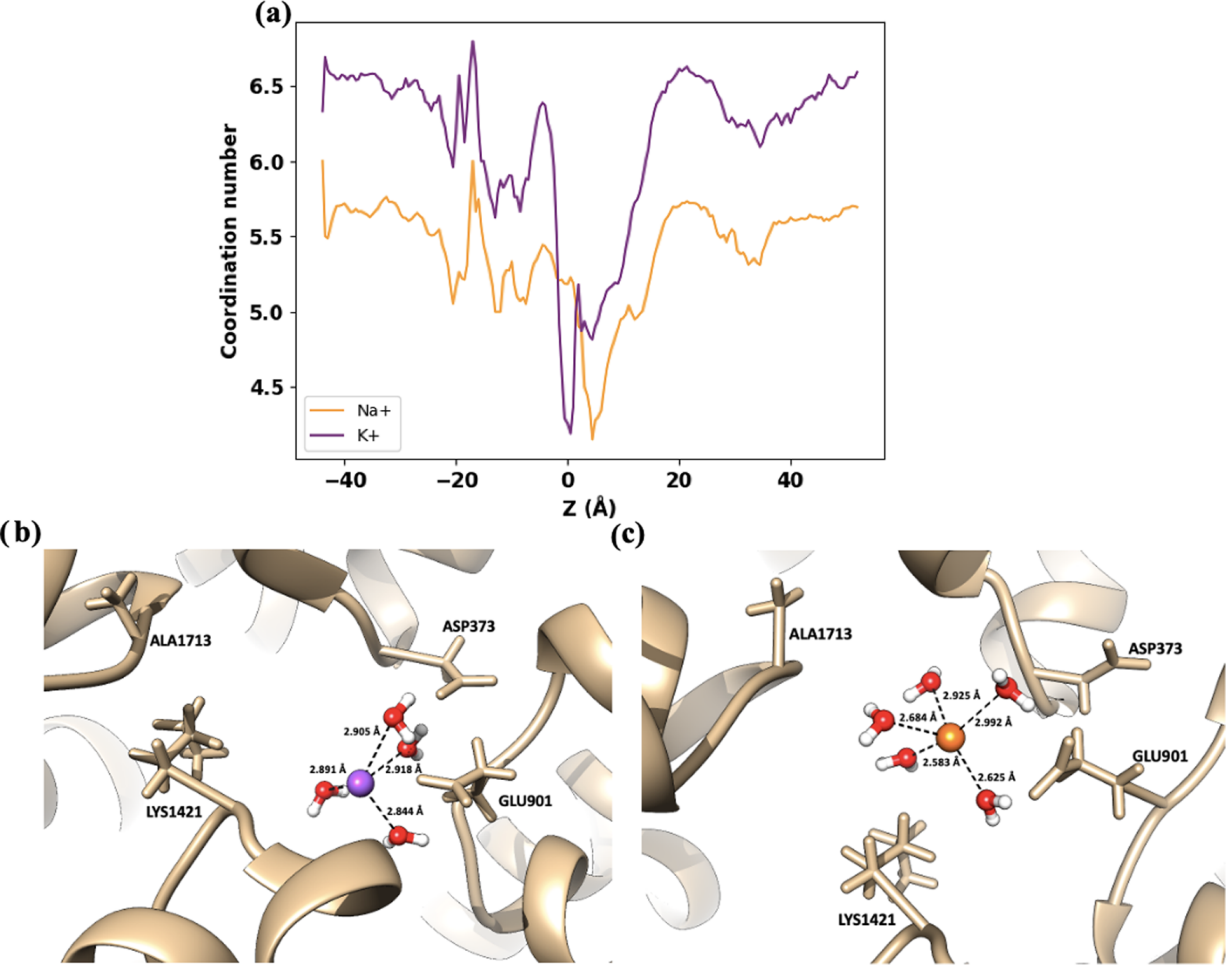
(a) Hydration numbers for Na^+^ and K^+^ ions along the *Z*-axis under a 1 V external field, showing differences in ion hydration as they move through the channel. Simulation snapshot of (a, b) potassium ion and (a, c) sodium ion at *Z* = 0 Å within the DEKA (SF) with their solvation spheres.

The water coordination numbers of Na^+^ and K^+^ were determined along the *Z*-axis based on the number of water–oxygen atoms within the coordination radius of each ion, and it is plotted in Figure 8a. The *Z*-axis was defined along the channel’s pore axis, running perpendicular to the membrane plane, with the origin (*Z* = 0) set at the geometric center of the DEKA motif. The coordination radius values were adopted from Corry and Thomas (Na^+^: 3.0 Å, K^+^: 3.4 Å).^13^ In the bulk solvent region, Na^+^ and K^+^ ions were coordinated with an average of 5.7 and 6.5 water molecules, respectively, consistent with previous studies.^13,17^ When the ions enter the channel and approach the EEDD ring (at around *Z* = 5–10 Å), they lose one water molecule from the solvation shell. The dehydration penalty is likely compensated by the attractive interactions with the four negative amino acids, consistent with the coordination analysis performed above. Then, when the K^+^ goes from EEDD to DEKA, it loses one additional water molecule to interact more effectively with the negative residues of the DEKA ring. However, Na^+^ is rehydrated, and it gains the water molecule that is lost at the beginning of the permeation process. The recovery of the solvation shell likely happens to compensate for the repulsion undergone with the positively charged lysine. Since the size of the Na^+^ ion is smaller than that of the K^+^ ions, reflected in the theoretical model by a smaller van der Waals radius, the positive charge concentration is larger, and the repulsion with lysine is stronger for Na^+^. The recovery of the solvation shell is likely a slow process, which retains the Na^+^ ion at the DEKA ring for a relatively long time, resulting in a deeper free energy minimum and a longer retention time.

Our simulations predict that the Na^+^ ions must be rehydrated when permeating close to the DEKA ring to avoid repulsion with the lysine amino acid. This would reinforce the importance of the lysine residue in the conduction mechanism observed in previous experimental studies.^7,21,22^ Moreover, this would also explain why mutation of this residue induces a loss of ion selectivity. However, the delicate energetic balance between the attractive energy gained when the solvation sphere is recovered at DEKA and the attractive and repulsive energy lost because of the larger distance between Na^+^ and the protein residues is not well described by the force field. This might be responsible for the deep energy minimum observed at DEKA that makes Na^+^ conduction less important than K^+^ conduction. Although it is likely that electrostatic interactions regulate ion permeation due to the presence of strong charges at the EEDD and DEKA rings, a possible role of polarization interactions cannot be ruled out. Thus, a parametrization of an improved force field, likely based on quantum mechanical calculations, would be necessary to describe the intricate situation predicted by our simulations. It is possible that this improved force field must include polarization effects. But even if this is not the case and a force field based on fixed point charges is appropriate, a reparametrization is necessary.

## Conclusions

Ion channels are crucial regulators of cellular electrical excitability, positioning them as key players in a broad array of physiological functions, including neuronal communication, muscle contraction, and cardiac rhythm regulation. In particular, Na_*V*_ channels are pivotal for modulating the Na^+^ flux across the cellular membrane, thereby facilitating the rapid depolarization necessary for the initiation and propagation of action potentials. For the hNa_*V*_1.5 channel, which is integral to cardiac physiology, a thorough understanding of its conductance and selectivity properties is essential for elucidating its role in normal cardiac function and the development of targeted therapies. Proper ion conduction is determined not only by the structural attributes of the channel but also by a delicate balance between the hydration state of the ions and their interactions with specific residues along the channel pore. These factors were computationally investigated in the present work by means of molecular dynamics simulations.

Our simulations predict that the conduction mechanism in the hNa_*V*_1.5 channel exhibits pronounced differences in the hydration and dehydration dynamics of Na^+^ and K^+^ ions as they traverse the EEDD and DEKA regions. In the EEDD ring, both ions lose one water molecule from their solvation shells, enabling strong, attractive interactions with the four negatively charged residues in this area. Contrary, the behavior of both ions at the DEKA ring is different, significantly influencing the ion conduction mechanism. Due to its smaller radius and higher charge density, Na^+^ tends to rehydrate in the DEKA region, possibly to avoid repulsion with the positively charged lysine residue, creating an energy well that temporarily traps the ion. Similarly, the importance of ion dehydration and rehydration is also observed in prokaryotic Na_*V*_ channels, such as Na_*V*_Ab,^13^ where ions must desolvate to permeate through the EEEE selectivity filter. This shared mechanistic step in both prokaryotic and eukaryotic channels underscores the fundamental role of solvation dynamics in ion conduction. However, unlike in eukaryotic channels, prokaryotic channels lack an additional extracellular ring, such as the EEDD ring, positioned above the selectivity filter. This distinction adds an extra layer of complexity to the conduction mechanism in eukaryotic channels, influencing both ion selectivity and conduction.

The depth of this energy well is overestimated by the force field employed in the simulations, impairing the conduction of Na^+^. Conversely, K^+^ undergoes continuous dehydration at the DEKA ring, allowing for a smoother passage through the filter without the need to re-establish its solvation shell. This difference in the hydration dynamics of Na^+^ and K^+^ highlights that ion selectivity is not solely dictated by direct ion–protein interactions but rather by the capacity of each ion to adapt its solvation state to the changing electrostatic environment imposed by the channel’s structural features. Such distinct solvation profiles are crucial in determining the energy barriers that each ion must overcome and play a definitive role in channel conductance and selectivity. Moreover, another source of error might be the poor sampling of the slow degrees of freedom inherent to conventional molecular dynamics simulations. Thus, ion hydration and potential conformational changes of the protein structure are likely not properly captured in our simulations. Enhanced sampling methods, such as metadynamics or umbrella sampling, could prove valuable in characterizing these slow processes and providing a more comprehensive understanding of the conduction mechanisms involved.

The inherent limitations of the employed classical force field, which is not able to describe ion selectivity in this channel, must be highlighted. We have used a conventional force field that describes the nonbonded electrostatic and nonelectrostatic interactions by fixed-charge Coulomb and Lennard-Jones potentials, respectively. It is well-known that such models do not properly describe polarizable effects and Pauli repulsion, two interaction types that might play an important role in the conduction mechanism. Further analyses based on quantum mechanical calculations would be needed to corroborate this conclusion. These shortcomings may lead to a wrong description of solvation and protein interactions, especially with the lysine residue and the associated energy barriers critical for ion transport through the channels. This underlines the necessity for applying more sophisticated approaches, such as molecular dynamics simulations based on polarizable force fields or quantum mechanical forces.
